# Pecan color change during storage: Kinetics and Modeling of the Processes

**DOI:** 10.1016/j.crfs.2022.01.015

**Published:** 2022-01-21

**Authors:** Himanshu Prabhakar, Clive H. Bock, William L. Kerr, Fanbin Kong

**Affiliations:** a Department of Food Science & Technology, University of Georgia, Athens, GA, USA; b USDA-ARS-SEFNTRL, Byron, GA, USA

**Keywords:** Quality, Predictive modeling, Post harvest, Activation energy, Pecan, Temperature, Relative humidity

## Abstract

Postharvest changes in pecan nutmeat color are affected by many factors, both internal and external. The temperature, relative humidity (RH) of the surrounding environment, and storage time are major factors contributing to color deterioration of the nutmeats. Kinetic models have long been employed to provide insights into the physical and chemical changes in food systems; however, no kinetic model has been developed describing the color changes of pecan nutmeats during storage. The objective of this research was to determine the effect of temperature, RH and storage time on pecan nutmeat color change. Pecan nutmeats of three commercially important cultivars (Stuart, Pawnee and Desirable) were subjected to different temperatures (20, 30 and 40 °C) and RH conditions (30, 50, 75% and 80%) for up to 450 days in simulated storage. The observed color changes of the pecan nutmeats were measured as lightness, chroma and hue (LCh). Additionally, the USDA pecan color rating scale was digitized to encourage its use among researchers. It was observed that the change in hue followed a zero-order decay whereas change in lightness and chroma followed a first-order decay. The value of the reaction constants ranged from 0.010 to 1.315 day^−1^. An Arrhenius model was used to estimate the activation energy (E_a_) corresponding to different storage conditions. The values revealed significant effects of temperature, RH and storage days on color degradation. The breakdown of flavonoids and reaction products from Maillard browning could be responsible for the formation of the reddish-brown color observed in degraded nutmeats. The kinetic parameters and models were used to develop a user-friendly online interface for predicting color change depending on selected parameters, with illustrations of the resulting pecan color (https://tinyurl.com/uspecans). The results of this study will aid pecan growers, processors and researchers to predict and visualize changes in color of pecan nutmeats during storage under various conditions of temperature and RH, and duration of storage. Although the study used cultivars Stuart, Pawnee and Desirable, the results likely have more general applicability to other cultivars too.

## Highlights

• A systematic investigation of the kinetics associated with pecan color change during storage was conducted.
• Temperature and relative humidity (RH) affected rate constants (k) and activation energy (E_a_) of pecan color.
• A digital version of the USDA pecan color scale was developed.
• An online model was built for pecan color prediction (https://tinyurl.com/uspecans).

## Graphical abstract

**Figure:**
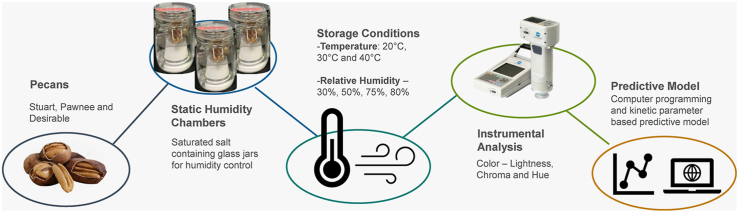

## 1 Introduction

*Carya illinoinensis* (Wangenh.) K. Koch, commonly known as pecan, is a native North American tree species cultivated across the southern states of the U.S. from Georgia to California. The U.S. is a major producer of pecans, and is responsible for 40–45% of the world's total pecan production (McEachern, 2014; NASS, 2020), making it a commercially important specialty crop in the U.S., with a market value of US$560 to 700 million (NASS, 2020). Worldwide, the U.S. has the largest per capita annual consumption of pecans (136–272 g) (Miaschi, 2018). Nonetheless, 45% of U.S. grown pecans are exported to other countries, with a value of more than US$470 million (ERS, 2021). Thus, the commodity is widely traded.

During domestic and international distribution and transportation, pecans may experience adverse environmental conditions which can cause quality losses. One of the chief quality losses to occur is color degradation. Indeed, a pecan nutmeat's color is used as an indicator of freshness and quality by wholesale distributors and retailers (Florkowski and Hubbard, 1994; S. J. Kays, 1979). Pale colors indicate freshness, while dark colors indicate advanced age and rancidity. Recently, the United States Department of Agriculture (USDA) updated the pecan color standard scale based on a study by Thompson et al. (1996). The study recommended a 6-color scale instead of 4-color scale to include a wider range of pecan color obtained after harvesting. The new scale, based on the Munsell system, embraces a wider range of pecan colors to represent that observed after harvesting. It comprises a 1 to 6 ordinal scale representing the colors light cream, cream, golden, light brown, reddish brown and dark reddish brown. The characteristic cream to golden color associated with fresh pecans is due to the presence of various phytochemicals including carotenoids and flavonoids (Kays and Wilson, 1976). As noted, with time, pecan color changes from bright cream, yellow or golden to either brown or reddish brown. The reason(s) behind the change in color with nutmeat degradation is not fully explored (Prabhakar et al., 2020). But considering the importance of nutmeat color, developing a dynamic understanding of the effect of environmental conditions and time based on models to predict nutmeat color change will aid growers, processors and retailers to better plan storage and distribution of the commodity.

Pecan nutmeats contain various macro and micronutrients that play a significant role in quality changes. Pecan nutmeats are rich in poly unsaturated fatty acids (PUFA), making them susceptible to lipid degradation (Polmann et al., 2019; Y. Zhang et al., 2018). Similarly, change in color may be due to various chemical reactions that occur in the nutmeats. The changes in color can be quantified using kinetic parameters including rate constants (k) and activation energies (E_a_). Kinetic parameters and rate constants are important components of kinetic models which can be used to explore reasons for changes in food quality and shelf life of food products (Haefner, 2005). Kinetic modeling aids in understanding the quality changes at a molecular level (Van Boekel, 2008). It is an established and reliable technique that has been used to predict quality changes in various food products, including almonds (Ciftci and Ozilgen, 2019), walnuts (Vinson and Cai, 2012; B. Zhang, Zheng, Zhou, Huang and Wang, 2016), soybean (Kong and Chang, 2009), various fruit (Aamir et al., 2013; Lin and Tiejin, 2001; Pereira et al., 2006; W. Zhang, Luo, Wang, Gu and Lv, 2021), hazelnuts (Özdemir and Devres, 2000), and many other commodities (Ayustaningwarno et al., 2020; Song et al., 2019). No kinetic models have been developed for pecans, despite pecan nutmeats being perishable, and despite the fact that they rank third among tree nuts in area under production in the U.S. – only almond and pistachio have larger production areas (NASS, 2017). During the postharvest process of storage, transport, redistribution, retail shelf time, and in some cases export, pecans experience a range of conditions in temperature and relative humidity (RH). Temperature may attain 50 °C and RH as high as 80%, respectively (Premtitikul, 2012), but most previous research has focused on storage temperatures ranging from 0 °C to 30 °C and an RH of 50–75%.

The overall objective of this study was to systematically investigate the effects of temperature (20, 30 and 40 °C), RH (30%, 50%, 75% and 80%), and storage time on pecan color change. The temperature based kinetic attributes, including the rate constants (k), activation energies (E_a_), and the ratio of rate constants for temperature increase of 10° (Q_10_ coefficients), were determined for pecan nutmeat color change. The kinetic attributes were subsequently used to design a user-friendly online interface that allows input of temperature, RH and storage time parameters to determine the impact on pecan nutmeat color.

## 2 Material and methods

### 2.1 Pecan production and source of nutmeats

Three cultivars of pecan (Stuart, Pawnee and Desirable) were harvested from orchards located at the USDA-Agriculture Research Service (ARS) Fruit and Tree Nut Research Laboratory, Byron, Georgia (U.S.A.), (+32.6650 N, + 83.7419 W, elevation of ≈156 m, 240 d freeze-free growing period, annual precipitation of 118 cm). Orchards received standard tree management practice for the state of Georgia (Wells et al., 2019). The experiment was performed twice, with pecans harvested in November 2018 and December 2019, respectively. In each season, the pecans were processed within 1 week of harvesting. The harvested pecans were conditioned prior to shelling by dipping in 85 °C water for 3 min, followed by drying at room temperature for 20–25 min and shelling via mechanical sheller (Modern Electronics, Mansfield, LA) (Forbus Jr and Senter, 1976). After shelling, pecans were dried at 20 °C and 45% RH overnight to a moisture content of 4–5% moisture content (AOAC, 2016) and stored at −20 °C in a commercial freezer until use in the experiments. Information on the different grades of pecans is provided (Table 1).

**Table 1:** Pecan samples used in the study. The classification system used to determine pecan quality was based on the USDA pecan quality standard (USDA, 1969). The pecan color grade was determined using the USDA pecan color rating scale (Fig. 3).

Year	Cultivar	Color grade	Nut Size (halves/kg)
2018	Stuart	Cream, No.2	Mammoth (≲ 550)
	Pawnee	Cream, No.2	Jumbo (662–770)
	Desirable	Cream, No.2	Mammoth (≲ 550)
2019	Stuart	Cream, No.2	Junior Mammoth (552–660)
	Pawnee	Cream, No.2	Mammoth (≲ 550)
	Desirable	Cream, No.2	Mammoth (≲ 550)

### 2.2 Experimental plan and design

**Fig. 1:**
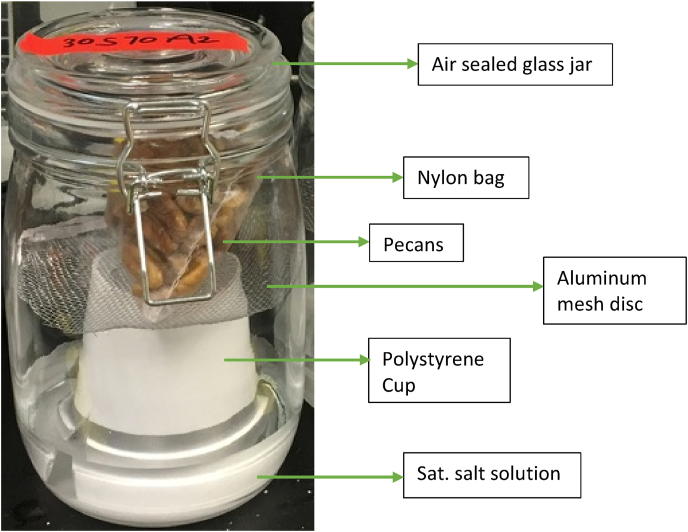
The static humidity chamber used in the pecan nutmeat storage experiments.

The desired RH was achieved by using 200 mL saturated salt solutions placed in a static humidity chamber (STC) which was a 1-L glass jar with a rubber gasket to seal the lid. The saturated salt solutions used to achieve the different RH were: magnesium chloride (30–32% RH), magnesium nitrate (50–52% RH), sodium chloride (75% RH) and ammonium sulfate (80–81% RH) (Certified ACS, Fisher Chemical, Waltham, MA). For the sake of simplicity, the RHs will be denoted as 30%, 50%, 75% and 80%, respectively, at each storage temperature. The STCs were placed in temperature-controlled chambers at 20, 30 and 40 °C. For each temperature × humidity treatment (replicates, n = 2), 50 g of whole pecans were placed in a nylon bag suspended above the saturated solutions on an aluminum mesh disc in the STC (Fig. 1). To simulate a real storage environment and corresponding air composition, the jars were opened periodically (every 1–2 weeks) for 30 s to allow fresh air into the container. The samples were drawn (n = 2) at predetermined intervals based on previous reports of pecan color change in the literature (Blackmon, 1932; Brison, 1945; S. Kays, 1979; Magnuson, Koppel, Reid, & IV, 2015; Mexis et al., 2009; Senter and Wilson, 1983). A total of five samples of nutmeats were collected from the STCs for each temperature × RH condition treatment (+1 baseline sample). The STCs were stored in duplicates (n = 2) for each treatment combination. The storage time ranged from 15 to 450 days, depending on the treatment (Table 2). The fungal growth assessment was done visually.

**Table 2:** Storage time in days for all environmental conditions used in the experiment (temperature and relative humidity). The storage matrix was the same for all cultivars (Stuart, Pawnee and Desirable) and crop years (2018 and 2019).

Temperature (°C)	Relative humidity (%)			
	30	50	70	80
**20**	450	380	225	40
**30**	380	230	150	25
**40**	300	150	100	15

The experimental design was a generalized randomized complete block design (GRCBD) with samples drawn on 5 occasions and 1 baseline or control sample (year and cultivar were treated as block effects). Storage time was unique to each RH condition at the different temperatures. Thus, storage time was nested within RH. A similar condition existed for cultivar and year with cultivar being nested within year. In the GRCBD, treatments (combinations of temperature, RH, and storage days) were replicated within each block (combination of year and cultivar). In an ordinary (unreplicated) randomized complete block design (RCBD), if there are insufficient degrees of freedom to fit block by treatment interaction terms it is necessary to assume that treatment effects do not differ across blocks (Shieh & Jan 2004). Such an assumption is often considered reasonable. In the current GRCBD, there is replication within each block, so block by treatment interactions could be included in the model and assessed. However, including such terms would make the model quite complex and cumbersome and the interpretation of the fitted model would become challenging. Thus, to simplify the analysis, the block by treatment interaction terms were omitted, as one would do for an ordinary, unreplicated RCBD.

### 2.3 Color measurement

A hand-held trismulus Minolta Chroma Meter (Minolta Corp., Ramsey, NJ) was used to measure the color characteristics of the pecan samples using the Hunter's L, C, and h scale, where L is lightness (0.00 = black to 100.00 = white), C is Chroma (0.00 = grey to 100.00 = bright or intense), and h is Hue (0–360°). Delta Hue (Δh), which is the difference in current hue and baseline hue, was also calculated as an alternative measure of hue. The observations were made under the International Commission on Illumination (CIE) standard illuminant D_65_, representing average daylight and color temperature of 6500 K. The colorimeter was calibrated using a white standard plate with the following color coordinates for lightness (L) = 97.59, green to red (a) = 0.39, and blue to yellow (b) = 1.75) before the color measurements of the pecan samples were taken. For color measurement, a uniform bed of pecan kernels was dorsally exposed to colorimeter lens. The measurements were done by taking 50 g of kernels and dividing into 3 groups (n = 3, pseudo-replicates), each containing approximately 5–8 pecans.

### 2.4 Kinetic analysis

To study the impact of storage period and temperature on pecan nutmeat color, kinetic parameters associated with the measured change in color were calculated. Chemical reaction kinetics can be applied to quantify color attributes of a food in the form of the general rate law (Lado and Yousef, 2002; Van Boekel, 2001):

(1) $$\frac{dP}{dt}=\pm kP^{n}$$

where *k* is the rate constant, *t* the reaction time, and *n* the reaction order. In general, *P* represents a quantitative value of L, C and/or h. According to Ling et al. (2015), the kinetics of food quality change follow zero (equation (2)) or first (equation (3)) order reactions:

(2) $$P=P_{0}-kt$$

(3) $$P=P_{0}e^{-kt}$$

An Arrhenius equation was used to investigate the effect of temperature (T) on the rate of color degradation (*k*):

(4) $$k=k_{0}e^{-\frac{E_{a}}{RT}}$$

where *k* is the rate constant, *k*_*0*_ is a pre-exponential factor, R is the ideal gas constant (8.314 J mol^−1^K^−1^), and T is the absolute temperature (K). E_a_ is the activation energy (J.mol^−1^) and is defined as the minimum energy needed to start a chemical reaction (sometimes called the energy barrier). Equation (4) can be rewritten as;

(5) $$\ln\;k=-\left(\frac{E_{a}}{R}\right)\left(\frac{1}{T}\right)+\ln\;k_{0}$$

By plotting ln *k* with 1/T, slope (E_a_/R) can be obtained which can be further solved to calculate E_a_. Chemical reactions are sensitive to temperature, and the Q_10_ value of a reaction is often used for reporting the temperature dependence and effect on the reaction rate as the temperature increases or decreases by 10 °C:

(6) $$Q_{10}=\frac{k_{2}}{k_{1}}$$

where *k*_1_ is reaction constant at temperature *T*_1_ and *k*_2_ is the reaction constant at temperature *T*_2_ (*T*_2_ = *T*_1_+10 °C). equation (5) and equation (6) can be combined to calculate Q_10_ as:

(7) $$Q_{10}=\frac{k_{2}}{k_{1}}=e^{\left(-\frac{E_{a}}{R}\right)\left(\frac{1}{T_{2}}-\frac{1}{T_{1}}\right)}$$

Q_10_ is a unitless value.

### 2.5 Development of a user interface for prediction of pecan nutmeat color based on storage conditions

The models and parameters derived from kinetic analysis were incorporated into a web application to provide a user interface that can be used to predict nutmeat color. The online application was constructed using computer programming languages including hyper Text Markup Language (HTML), javascript and Cascading Style Sheets (CSS). A similar program was constructed for Microsoft Excel which is downloadable and does not require internet access to operate. For Excel, inbuilt functions including *IF*, *IFERROR*, *AND* and *OR* were used to make a prediction statements from inputs provided by the user. The web application is compatible with most commonly available web browsers and devices.

### 2.6 Statistical analysis

Based on an inspection of the normal probability and quantile plots of the data and the observation that the variance was approximately constant throughout the distribution, it was concluded the data were amenable to parametric analyses. However, distribution of storage days was found to be right skewed. Thus, storage days were transformed by taking Log (Storage days + 1). For instance, transformed storage day values of 0, 20, and 30 days were 0, 1.32, and 1.49 days, respectively.

First, a mixed model analysis was used to determine the effects of temperature, RH and storage time on color attributes (LCh). Storage days, temperature (°C), and RH (%) were considered fixed effects whereas crop year and cultivar (which was nested in crop year) were treated as random effects. The two-way interactions among fixed effects (temperature, RH and storage days) were studied, as were the two-way interactions where storage days were nested within RH. A Tukey's HSD *post hoc* test (ɑ = 95%) was performed to explore whether there were differences among means for the different treatments. The data recorded from any specific treatment or RH was independent of other treatments and was not affected by pecan sampling. The treatment results are presented as the mean± the standard deviation (SD). The effect of cultivars on color attributes was not studied due to limitations of experiment design, and the need for more data from a wider range of cultivars and years to draw firm conclusions (thus, cultivar data was pooled in the mixed model analysis).

Second, linear and exponential regression analyses were used to determine the rate constants (*k*) and activation energy (E_a_). A linear regression analysis was used to determine *k* and E_a_ for hue (independent variable) and storage days (dependent variable, not transformed). An exponential model was used to calculate *k* and E_a_ for lightness and chroma (both independent variables) vs storage days (dependent variable, not transformed). The fit for both models were assessed based on the adjusted coefficient of determination (adj. R^2^). All the statistical analyses were performed using JMP®, Version 15 Pro (SAS Institute Inc., Cary, NC).

## 3 Results

### 3.1 Pecan color changes during storage

**Fig. 2:**
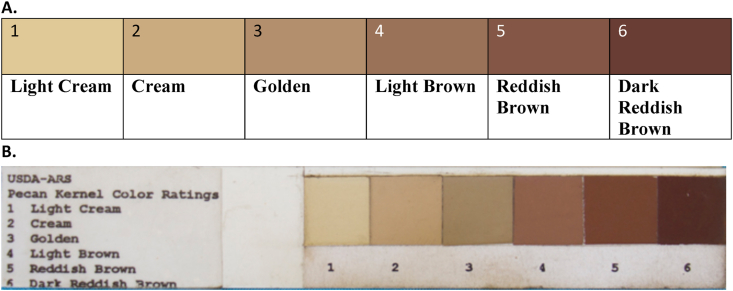
The digitized version of the pecan color scale and standards (A) adapted from the USDA pecan color rating scale (B) and results presented by Thompson et al. (1996). The digital version of the scale can be reproduced anywhere using the color coordinates provided in Table 3. (For interpretation of the references to color in this figure legend, the reader is referred to the Web version of this article.)

A digitized version of the USDA 6-point pecan nutmeat color scale was created (Fig. 2), and the color coordinates of the Thompson et al. (1996) pecan nutmeat color scale was tabulated (Table 3). The pecans harvested at the end of the 2018 and 2019 crop seasons (from all three cultivars) and used for storage experiment were of a cream color grade on the USDA pecan color scale (color grade 2) (Table 1).

**Table 3:** The color co-ordinates corresponding to the six grades of the USDA pecan color rating scale (Thompson et al., 1996; USDA, 1969).

Grades	Color coordinates^a^			
	HVC	RGB	Lab	LCh
**Light cream**	2.5Y,8,4	223,200,150	81,1,28	81,28,88°
**Cream**	10 YR, 7,4	202,171,128	72,5,27	72,27,79°
**Golden**	7.5 YR,6,4	179,142,110	62,9,22	62,24,67°
**Light brown**	5 YR,5,4	154,115,89	52,12,21	52,24,60°
**Reddish brown**	2.5 YR,4,4	130,87,69	41,16,18	41,24,49°
**Dark reddish brown**	10 R,3,4	105,61,52	31,18,14	31,23,37°

a HVC- (Hue, Value, Chroma), RGB (Red, Green, Blue), Lab (lightness, red to green, blue to yellow), LCh (lightness, Chroma, Hue), Y (Yellow), YR (yellow-red), R (Red).

The summary of the mixed model analysis of main effects, and two-way interactions affecting color attributes (L, C and h) showed that all main effects, including RH (a factor whose effect on pecan color has not been studied previously), and interactions were significant for each color attribute (Supplementary Table S1). Based on Tukey's means separation, all color attribute values showed a decline with storage days at the various temperatures and RH combinations used in the experiment (Supplementary Tables S2–S5).

**Fig. 3:**
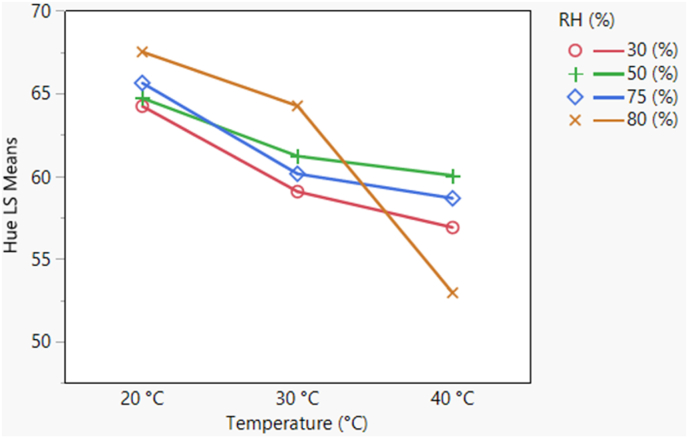
Interaction plot illustrating change in pecan hue with changes in relative humidity (%) and temperature (°C). Note that hue values approaching zero indicates red color (F-ratio = 8.34, p-value <0.05). (For interpretation of the references to color in this figure legend, the reader is referred to the Web version of this article.)

The interaction between temperature and RH is shown in Fig. 3. At 20 °C, rate of color degradation was relatively less over the storage days. But as temperature increased, hue declined considerably. At a RH of 50% or less, the hue values indicated development of red color. In contrast, at 75% and 80% RH, change in hue was greater. Thus, color change was found to be dependent on varying temperature and RH conditions. According to the LCh color scale, hue values approaching zero corresponds to a red color and whereas high hue values (approx. 65°–60°) corresponds to a brown color. The interaction between temperature, RH and storage days shows a change in slope with storage days as storage conditions change. The slope becomes steeper as temperature and RH increase.

**Fig. 4:**
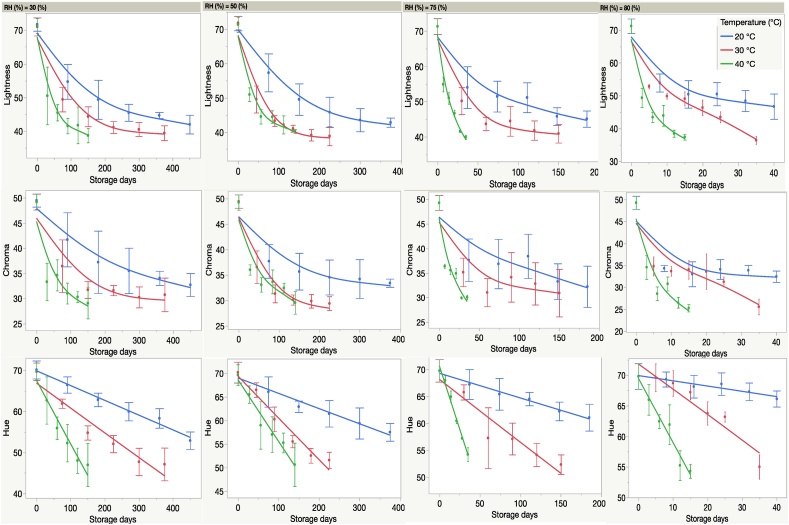
The change in color attributes of pecan nutmeats during storage periods of up to 450 days at different temperatures and relative humidities. The lightness and chroma of pecan color followed a curvilinear (exponential) decay whereas hue followed linear decay during storage. The error bars represent the standard deviations of the mean values based on 12 replicates. (For interpretation of the references to color in this figure legend, the reader is referred to the Web version of this article.)

The regression analyses of the individual color attributes, L, C and h, showed different patterns of decline over time, depending on temperature and RH (Fig. 4). The lightness and chroma presented an exponential decay during storage whereas hue followed a linear decay. The rate of color darkening increased at higher temperature; the slope for change in hue was steeper and decreased linearly with storage time. For each increase in temperature, the slope was steeper, indicating a zero-order decay. For lightness and chroma, the relationship was curvilinear, indicating that color attribute degradation became stagnant after an initial sharp decrease, indicating a first-order decay. The mean Δh (an alternative measure of ΔE) at each time point, temperature and RH is presented and decreased in a constant manner regardless of cultivar (Supplementary Tables S5 and S6).

### 3.2 Kinetic analysis of color degradation

The rate constants (k) for lightness, chroma and hue increased with temperature for all three cultivars (Table 4), and the extent of variability in color attributes in the different harvesting seasons can be seen. All reported *k* values are negative, denoting declining slope. The activation energy, E_a_ was derived using an Arrhenius model, and the range in E_a_ values for hue (h), lightness (L) and chroma (C) were: 20.08–57.30 kJ/mol, 6.53–25.38 kJ/mol, and 7.63–28.05 kJ/mol, respectively. The *k* and regression parameters of all color attributes are also provided (Table 4). Decline in hue was more temperature sensitive compared to the decline in lightness or chroma.

**Table 4:** Summary of the rate constants (k) and activation energy (E_a_) for color attributes pertaining to pecan nutmeat color of three different cultivars (Desirable, Pawnee, and Stuart) stored at different temperatures and relative humidities (replicates, n = 2).

Cultivars	Temp (°C)	RH (%)	Lightness			Hue			Chroma		
			k (day^−1^)	Adj. R^2^	E_a_ (kJ/mol)	k (day^−1^)	Adj. R^2^	E_a_ (kJ/mol)	k (day^−1^)	Adj. R^2^	E_a_ (kJ/mol)
**Desirable**	**20**	**30**	0.006 ± 0.001	0.85	15.10 ± 13.52a	0.032 ± 0.001	0.95	29.16 ± 8.08 b	0.001 ± 0.003	0.68	23.83 ± 14.89a
	**30**		0.012 ± 0.008	0.98		0.066 ± 0.001	0.87		0.011 ± 0.006	0.91	
	**40**		0.019±0.023	0.89		0.157 ± 0.037	0.91		0.025 ± 0.034	0.85	
	**20**	**50**	0.013 ± 0.003	0.94	11.33 ± 16.02a	0.033 ± 0.006	0.78	30.34 ± 3.75 b	0.018 ± 0.013	0.8	16.23 ± 22.95a
	**30**		0.021 ± 0.001	0.98		0.082 ± 0.012	0.93		0.017 ± 0.012	0.99	
	**40**		0.040 ± 0.009	0.94		0.178 ± 0.019	0.77		0.047 ± 0.007	0.9	
	**20**	**75**	0.041 ± 0.001	0.94	12.64 ± 6.65a	0.045 ± 0.009	0.82	40.87 ± 1.25 ab	0.053 ± 0.002	0.85	22.60 ± 5.94a
	**30**		0.039 ± 0.043	0.92		0.164 ± 0.016	0.86		0.077 ± 0.002	0.77	
	**40**		0.061 ± 0.020	0.97		0.474 ± 0.021	0.97		0.105 ± 0.12	0,92	
	**20**	**80**	0.156 ± 0.116	0.95	10.77 ± 12.96a	0.050 ± 0.017	0.52	57.30 ± 7.78a	0.268 ± 0.005	0.89	24.86 ± 4.56a
	**30**		0.118 ± 0.166	0.94		0.324 ± 0.028	0.89		0.209 ± 0.247	0.93	
	**40**		0.348 ± 0.076	0.96		1.048 ± 0.175	0.96		0.402 ± 0.013	0.95	
**Pawnee**	**20**	**30**	0.010 ± 0.003	0.89	22.10 ± 0.19a	0.039 ± 0.005	0.85	20.08 ± 1.69 b	0.008 ± 0.009	0.63	23.32 ± 8.24a
	**30**		0.014 ± 0.006	0.93		0.052 ± 0.006	0.76		0.016 ± 0.021	0.81	
	**40**		0.017 ± 0.010	0.88		0.140 ± 0.021	0.83		0.040 ± 0.011	0.86	
	**20**	**50**	0.006 ± 0.006	0.97	15.27 ± 6.76a	0.027 ± 0.005	0.78	27.51 ± 4.77 ab	0.014 ± 0.002	0.92	12.81 ± 1.38a
	**30**		0.031 ± 0.002	0.99		0.081 ± 0.007	0.85		0.035 ± 0.002	0.99	
	**40**		0.039 ± 0.002	0.99		0.127 ± 0.014	0.97		0.053 ± 0.010	0.99	
	**20**	**75**	0.043 ± 0.001	0.86	10.26 ± 0.29a	0.054 ± 0.007	0.92	37.61 ± 0.92a	0.069 ± 0.020	0.82	14.89 ± 6.77a
	**30**		0.060 ± 0.028	0.93		0.130 ± 0.020	0.86		0.082 ± 0.013	0.66	
	**40**		0.064 ± 0.005	0.97		0.492 ± 0.005	0.99		0.110 ± 0.014	0.92	
	**20**	**80**	0.153 ± 0.005	0.82	18.00 ± 0.71a	0.171 ± 0.018	0.97	37.44 ± 3.83a	0.243 ± 0.058	0.95	8.70 ± 6.74a
	**30**		0.231 ± 0.034	0.97		0.312 ± 0.003	0.9		0.351 ± 0.031	0.95	
	**40**		0.412 ± 0.122	0.95		0.959 ± 0.121	0.84		0.385 ± 0.051	0.94	
**Stuart**	**20**	**30**	0.012 ± 0.001	0.99	19.55 ± 14.34a	0.043 ± 0.006	0.99	26.19 ± 3.75bc	0.010 ± 0.005	0.88	26.08 ± 26.57a
	**30**		0.020 ± 0.007	0.98		0.067 ± 0.011	0.93		0.024 ± 0.016	0.93	
	**40**		0.029 ± 0.025	0.95		0.166 ± 0.016	0.92		0.042 ± 0.036	0.95	
	**20**	**50**	0.008 ± 0.001	0.88	19.38 ± 2.16a	0.039 ± 0.005	0.88	22.28 ± 4.90c	0.021 ± 0.003	0.76	25.87 ± 13.87a
	**30**		0.023 ± 0.008	0.96		0.087 ± 0.014	0.87		0.024 ± 0.012	0.92	
	**40**		0.041 ± 0.004	0.97		0.130 ± 0.006	0.96		0.039 ± 0.042	0.9	
	**20**	**75**	0.013 ± 0.013	0.89	25.38 ± 20.41a	0.045 ± 0.011	0.8	42.75 ± 5.41 ab	0.034 ± 0.007	0.59	28.05 ± 1.16a
	**30**		0.037 ± 0.007	0.97		0.122 ± 0.001	0.86		0.042 ± 0.046	0.87	
	**40**		0.100 ± 0.064	0.97		0.489 ± 0.040	0.99		0.153 ± 0.031	0.93	
	**20**	**80**	0.182 ± 0.018	0.91	6.53 ± 3.14a	0.064 ± 0.022	0.18	46.44 ± 1.15a	0.386 ± 0.006	0.95	7.63 ± 1.40a
	**30**		0.261 ± 0.037	0.94		0.325 ± 0.039	0.95		0.373 ± 0.030	0.9	
	**40**		0.287 ± 0.036	0.97		1.315 ± 0.103	0.95		0.431 ± 0.025	0.94	

The values are tabulated as mean ± standard deviation (n = 2). k (rate constant), E_a_ (Activation Energy). E_a_'s with different letters indicate significant difference between estimated values (along column) based on Tukey's HSD (α = 0.05).

To examine the effect of RH on the rate of color degradation of pecan nutmeats, the activation energy (E_a_) was calculated and compared to all color attributes (LCh) across the range of RH. RH had a significant effect on E_a_ for hue (p < 0.05). The pecan nutmeats stored below 75% RH had significantly lower E_a_ for hue than pecans stored above 70% RH. Contrary to this, there was no evidence that RH had any significant effect on the E_a_ for either the lightness or chroma color attributes. Our experiment data was variable as evidenced by the large standard deviations in E_a_ for lightness and chroma, which may have contributed to the challenge of detecting differences for the two attributes (Table 4). Q_10_ values pertaining to the different storage conditions are presented in Table 5 and can be used to determine the extent of color change in pecans under various environmental conditions.

**Table 5:** Summary of Q_10_ values (hue only) of pecan nutmeats exposed to different temperatures.

Temperature	RH	Q_10_ value^a^
20 → 30	30	1.65 ± 0.37
	50	2.57 ± 0.39
	75	2.92 ± 0.64
	80	4.61 ± 2.39
30 → 40	30	2.52 ± 0.16
	50	1.74 ± 0.37
	70	3.56 ± 0.59
	80	3.45 ± 0.52

The values are tabulated as mean ± standard deviation (n = 2).

### 3.3 Predicting pecan color change during storage using kinetic parameters

One way to predict color change in pecan nutmeats is to identify the extent of color degradation represented by the rate constant (k). By checking the color grade of harvested pecans using the USDA pecan color scale (Fig. 2), the corresponding hue value can be determined (Table 3). For example, golden pecans (color grade no. 2) may have hue values ranging from 67° to 79° (Table 3). One approach is to take the average of these numbers, i.e. 73°. Assuming that the target pecan color grade is light brown (color grade no. 4), or a hue of 60°, the number of days it will take for the hue to change (under defined conditions) can be estimated. Albeit this is a rapid method to determine the initial hue value of pecan, and it is not as accurate as a colorimeter. A second approach is to determine color coordinates by taking RGB (Red Green Blue) coordinates. These coordinates can be acquired from a digital image of the pecan nutmeat by using commonly available computer software and online applications including MS Word and Google Sheets, respectively. The RGB coordinates can be further transformed to get lightness, chroma and hue values either using formulae or online color coordinate transformation web application(s).

The hue value obtained from either of the abovementioned two methods can be used in Equation (7):

(8) $$S=\frac{Initial\;hue-Final\;hue}{k_{T,RH}}$$

where S is number of storage days required for the pecan nutmeats to reach a final hue value, hue is color attribute value and k corresponds to the rate constant at the specified temperature (T °C) and relative humidity (RH %) (Table 4 should be used for locating the appropriate rate constant for k under specific storage temperatures and RH). Note that the pecan color prediction was based on the hue (h) attribute alone as it was found to be a reliable indicator for color change for most of the storage experiment conditions. The hue values obtained can be looked up on the Munsell color scale to obtain visual color.

A third method to determine color change is to use the E_a_ and Q_10_ values. The color prediction may be interpolated depending on the storage temperature by estimating rate constants (k), which can be derived from the activation energy (E_a_). Referring to Equation (5), *k*_*1*_ corresponds to the reaction rate for color degradation at the lower storage temperature (*T*_1_) and *k*_*2*_ corresponds to the reaction rate for color degradation at the higher storage temperature (*T*_2_). The values can be obtained from Table 4. The new *k* values can be used to calculate *S* in equation (7). A fourth, and quick way of determining *S* is to use the Q_10_ values presented in Table 5. The Q_10_ value represents an increase or decrease in the rate of a reaction with every 10 °C change in temperature. By dividing *S* with the Q_10_ value at a specific temperature (T), the new *S* can be determined at T+10 °C.

(9) $$S_{T+10{}^{\circ}C}=\frac{S_{T}}{Q_{10\left(T+10{}^{\circ}C\right)}}$$

For example, at 20 °C, 30% RH, and after 270 days in storage, pecan nutmeats developed a hue value of 60°. If the Q_10_ value for a temperature increase from 20 °C to 30 °C is 2.50, pecan nutmeats will develop a hue value of 60° in 108 days (i.e., 270 days/2.50) at 30 °C and 30% RH. These formulae were used to create the web-based application presented in the next section.

### 3.4 Web-based application to determine pecan nutmeat color change

**Fig. 5:**
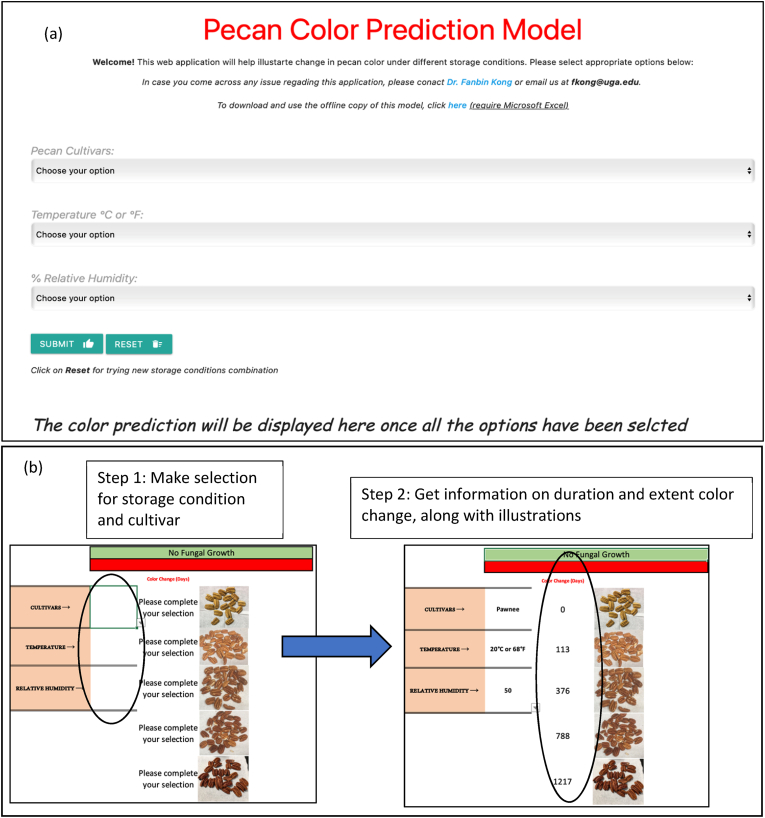
(a) Online web application for pecan color prediction - the user can select different storage condition combinations (cultivar, storage temperature and relative humidity) (b) the Microsoft Excel – version of the pecan color prediction model which can be downloaded to devices. The prediction from both these models will provide illustrations of pecan color change. (For interpretation of the references to color in this figure legend, the reader is referred to the Web version of this article.)

The rationale behind developing an online model to predict pecan color change under different storage conditions was to make the information accessible to a broad group, including other researchers and non-scientific audiences. The equations, constants and kinetic parameters reported above were incorporated into an easy-to-use interface where an individual can ascertain the impact of storage period, temperature, and RH on pecan nutmeat color (Fig. 5). The link to the web application can be found here (https://tinyurl.com/uspecans). An individual can obtain information on the effects of storage time and the extent to which color change will occur along with illustrations. The user can make a selection by clicking the down arrow (highlighted in Fig. 5). The down arrow activates a drop-down menu where the user can select the options for cultivar, temperature and RH. The results of the selection will show the number of days the pecan nutmeats will take to degrade. The users can refer to the USDA pecan nutmeat color scale to determining the grade of the pecan at different points in time during storage. The baseline color grade for the pecans used in the experiment was cream (color grade no. 2). So, the results may vary if the initial pecan nutmeat color grade increases or decreases. The use of the online model is not limited to any device and does not require installation of any software.

## 4 Discussion

High temperature (>30°) storage conditions resulted in a more dramatic decline in hue values as compared to storage at 20 °C. After 120 and 30 days, visual fungal growth and decay precluded color assessment of pecans stored at RH 75% and 80% (20 and 30 °C), respectively. No visual fungal growth was observed in pecans stored at 40 °C (30%–80% RH) until the end of storage period. Please note that fungal growth was not assessed microbiological and only visual observations were taken. The impact of change in chroma during storage on pecan color was minimal, hence the discussion pertaining to this color attribute is limited. Previous studies indicated that decline in lightness (L) of pecan nutmeats followed a linear trend (Forbus Jr. et al., 1980; Senter and Wilson, 1983). But we observed that lightness (L) exhibited a first order or exponential decay during long term storage. The previous storage studies were conducted over a maximum of 15 weeks, which could be the reason for the reported trend as linear if only the initial steep decline in L was captured. The effect of RH on color of pecan nutmeats was reported for the first time, although the effect was conditioned by temperature. Increase in RH of storage environment escalated the rate of color degradation, evident from increase in rate of reaction (k) and activation energies (E_a_).

**Fig. 6:**
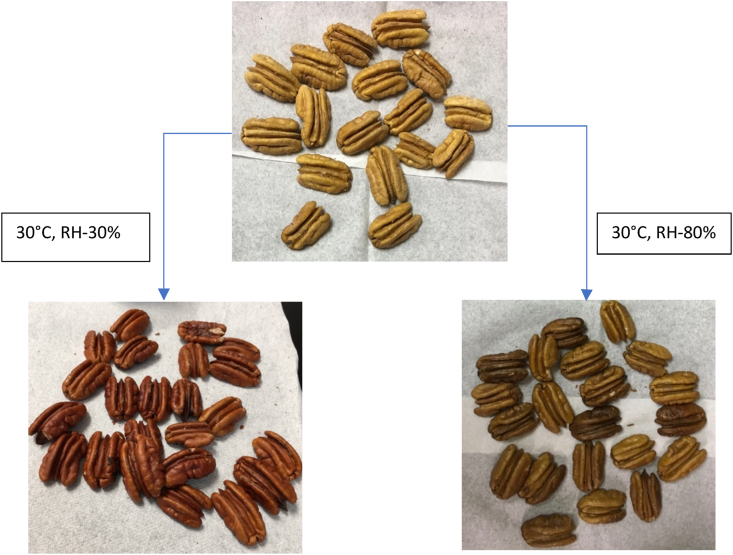
Color development in pecan nutmeats (cultivar Stuart) stored under different % relative humidity (30 and 80%) and at 30 °C. (For interpretation of the references to color in this figure legend, the reader is referred to the Web version of this article.)

These results are in agreement with those reported by Brison (1945), Kays and Wilson (1976) and Wright (1950). Development of the red and brown colors in pecan nutmeats with storage time is likely due to flavonoid degradation and the Maillard reaction (Senter et al., 1978). Flavonoids are one of the most common pigments in pecan nutmeats. Flavonoid molecules becomes very unstable if they come in contact with air and show affinity for polymerization when exposed to acidic conditions. Senter et al. (1978) conducted an extensive study identifying the flavonoids present in pecan nutmeats, namely leucoanthocyanidin and leucodelphinidin (flavan-3,4-diols). They exposed pecan nutmeats to 70 °C for 10 days and extracted the red colored condensed tannins (phlobaphenes and anthocyanidins). After extraction of the red colored pigments, the pecans were reported to be a light golden color, indicating the effect of a high concentration of the polymerized flavonoids on pecan nutmeat color. Rosenheim (1920) described the original unchanged flavonoid molecules as colorless. A study on discoloration of pears found that under similar temperature conditions, leucoanthocyanidins, along with catechin, underwent oxidation and polymerized resulting in red-colored condensed tannins, namely phlobaphenes and anthocyanidins (Springob et al., 2003). Pecans also possess flavan-3-ols in the form of catechins (de la Rosa et al., 2019). Whether catechins are involved in the phlobaphene formation in pecan nutmeats has not been established. In addition, the mechanism behind conversion of leucodelphinidin to condensed tannins is unexplored. The transformation normally happens in the presence of heat and under acidic conditions. During pecan storage, especially at high temperature (>25 °C), synthesis of high levels of primary and secondary oxidation products have been noted. The products of oxidation contribute to the acidity in the pecan nutmeat matrix (Magnuson et al., 2015; Pyriadi and Mason, 1968; Rudolph et al., 1992). The hot, acidic conditions, along with presence of oxygen in storage environment, is likely sufficient to initiate the chemical reactions that result in formation of the red color in pecan nutmeats (Fig. 6) (Ribeiro et al., 2020).

The brown color development in pecan nutmeats may be due to the Maillard reaction. Pecan is known for its high fat content, but it also contains a significant amount of sugar and protein (USDA, 2019). Sugar and protein are primary reactants for the Maillard reaction; the reducing end of the carbohydrate reacts with the –NH_2_ group of amino acids to form a Schiff base which undergo an Amadori rearrangement and degradation to form Maillard reaction products. The increase in temperature accelerates formation of Maillard reaction products, evident from changes in the activation energy (E_a_). In terms of food quality, E_a_ indicates the sensitivity of the reaction to changes in temperature. The range of reported E_a_ based on color change due to Maillard browning is 23–65 kJ/mol (Van Boekel, 2001). During the storage experiments, an increase in E_a_ was observed as the RH increased (Table 4). According to Labuza (1980), water activity and excess moisture content in food systems affects the rate of the Maillard reaction. The water activity, a_w,_ for stored pecans ranged from 0.43 (at RH of 30%) to 0.75 (at RH of 80%). Pecans stored at 20 °C and 70% or at higher RH (>70%) experienced rapid development of a brown coloration with all three cultivars. As temperature and RH increased to 40 °C and 80% RH, respectively, a reddish-brown color developed indicating the presence of reaction products of flavonoid polymerization and non-enzymatic or Maillard browning. Some reports suggest that the change in color of pecan nutmeats is due to lipid oxidation but it has not been experimentally established (Brison, 1945; Thewes et al., 2021). It could be argued that lipid oxidation indirectly affects the color development by providing suitable conditions for polymerization of flavonoids.

Conventionally, total color change (ΔE) has been used as a general parameter in foods to measure and understand overall change in color. However, it was observed that ΔE was not sensitive enough to describe changes in overall color of pecan nutmeats over the duration of the experiments. The lightness and chroma values decreased rapidly during the initial storage period and became stagnant afterwards. Contrary to this, hue values decreased steadily, which is why change in hue (denoted as Δh) could be employed to evaluate pecan nutmeat color change. The hue was found to be a good overall instrumental measurement parameter for pecan color but as RH increased (>75%), change in lightness became more prominent than change in hue. ΔL could be a reliable indicator of color change for pecan nutmeats stored at ≥75% RH. However, storing pecans at RH greater than 75% is not advisable as the pecans become susceptible to fungal growth and are thus rendered unsafe for human consumption.

Finally, a digitized version of the USDA 6-point pecan nutmeat color scale was created (Fig. 2). The USDA established the 6-point LCh pecan nutmeat color standard scale used by growers and processors to grade harvested pecans (Thompson et al., 1996), which is accessible to all researchers and pecan growers only through the USDA. Many researchers have relied on the Munsell Color or CIE systems to evaluate pecan nutmeat color (Kays and Wilson, 1977; Kays, 1979; Senter and Wilson, 1983; Von Wandruszka, Smith and Kays, 1980). Based on the results of Thompson et al. (1996), the digitized version of the USDA scale was created. Even though the USDA pecan nutmeat color scale was designed for grading pecans at the time of harvest, it is well suited to address the color changes in pecan during storage. Better accessibility and use of the color scale for reporting scientific data will bring greater uniformity and coherence in the narration of research reporting and other practical aspects of judging pecan nutmeat quality of U.S. grown pecans (the color coordinates given by Thompson et al. (1996) pecan nutmeat color scale has been tabulated in Table 3). For instance, it took approximately 270–300 days for pecan nutmeats to become light brown (color grade no. 4) at 20 °C and 30% RH. The pecan nutmeats attained color grade no. 4 more rapidly at higher temperature; the nutmeats took 124–157 days to turn light brown at 30 °C and 30% RH, but only 13–21 days to turn light brown at 40 °C and 30% RH, respectively. Ideally, perfectly ripened pecans possess a light cream color (color grade no. 1) but there may be anomalies. The baseline color grade for pecans used in this storage study was cream (color grade no. 2). It may take longer for pecan nutmeats of color grade no. 1 to degrade to color grade no. 6 of the USDA pecan nutmeat color scale. However, our model would be advantageous as it provides a conservative prediction on pecan nutmeat color.

The equations used for the online color prediction tool have some caveats. Even though the model predicts color change of pecan nutmeats, the current version can be used only for predicting the color of pecans stored under the range of experimental conditions in which the study was conducted (temperature, RH and storage time). Future studies that encompass additional factors and conditions (temperature, RH, packaging material, atmospheric composition etc.) will result in more information that can be integrated into the models to improve the usefulness, application, comprehensiveness and accuracy of such online tool. Additionally, the kinetic parameters can be readily modified to suit various conditions including improving farming practices, changing soil composition and introduction of improved cultivars.

## 5 Conclusion

Pecan nutmeat color is widely used as an indicator of freshness and quality among wholesale distributors and retailers. The rate of color degradation depends on storage conditions, particularly temperature and relative humidity. Pecans maintained under low humidity conditions (<75%) and high temperature (>30 °C) resulted in more rapid development of a reddish-brown color, possibly due to breakdown of flavonoids and Maillard reaction products. The remining conditions tested (RH>75% and temperatures of 20–30 °C) resulted in only a brownish pigment being detected - likely a result of the Maillard reaction alone. The pecan nutmeats experienced least color degradation during low temperature (20 °C) and dry environment (30% and 50% RH) conditions. The overall quality degradation of pecans (texture and oil quality) under these conditions needs further investigation. The difference in rate of decay diminished as storage temperature increased. The change in hue followed a zero-order reaction whereas lightness and chroma followed first-order decay. Kinetic parameters including rate constants, activation energy and Q_10_ values were calculated and incorporated in an online, easy-to-use interface where individuals can check the impact of storage, temperature and RH on pecan color (https://tinyurl.com/uspecans). Our experiments revealed that RH had a significant effect on activation energy (E_a_); E_a_ increased with an increase in RH indicating that the rate of color degradation (*k*) was elevated at high RH. By incorporating the constants and equations behind a user-friendly interface, a coherent system was constructed that simplified use of the research outcomes for non-scientific readers, pecan growers, and processors, and other stakeholders. Thus, our work highlights the possibility of integration of computer programming tools in food science research to build intelligible and practical models to predict quality changes in food.

## CRediT authorship contribution statement

**Himanshu Prabhakar:** experimentation and data collection, Formal analysis, writing. **Clive H. Bock:** statistical analysis, Supervision, writing. **William L. Kerr:** instrumental analysis, Supervision, writing and editing. **Fanbin Kong:** Conceptualization, of the experiment and experimental design, Supervision, writing.

## Declaration of competing interest

The authors declare that they have no known competing financial interests or personal relationships that could have appeared to influence the work reported in this paper.
