# Supplementary material for: Pecan color change during storage: Kinetics and Modeling of the Processes
Source: Curr Res Food Sci. 2022 Jan 21;5:261–71. doi: 10.1016/j.crfs.2022.01.015 (PMC8802062; doi:10.1016/j.crfs.2022.01.015)
Supplement: Multimedia component 1 [file mmc1.docx]

**Supplementary Information**

**PECAN COLOR CHANGE DURING STORAGE: KINETICS AND MODELING OF THE PROCESSES**

Himanshu Prabhakar^a^, Fanbin Kong^a*^, Clive H. Bock^b^, William L. Kerr^a^

*^a^ Department of Food Science & Technology, University of Georgia, Athens, GA, US.*

*^b^ USDA-ARS-SEFNTRL, Byron, GA, USA*

*Corresponding author: fkong@uga.edu

Supplementary Table S1 Summary of fixed effects and interactions of the mixed model analysis for hue lightness and chroma for pecans stored at different temperatures, relative humidities and storage days. The factor ‘storage days’ is nested within RH. The model’s adjusted coefficient of determination (R^2^) for hue, lightness and chroma were 0.77, 0.93 and 0.85, respectively. All of fixed effects and interactions were found to be significant at a 95% confidence level. Cultivar (nested in crop year) and crop year was blocking variable and were treated as random effects for the analysis.

| **Source** | **F Ratio (Hue)** | **F Ratio (Lightness)** | **F Ratio (Chroma)** | **Prob > F** |
| --- | --- | --- | --- | --- |
| **Temperature (T, °C)** | 133.72 | 320.96 | 180.96 | <0.05 |
| **RH (%)** | 5.18 | 222.76 | 183.58 | <0.05 |
| **Storage days [% RH]** | 213.84 | 1184.53 | 545.06 | <0.05 |
| **T * RH** | 8.34 | 22.52 | 13.47 | <0.05 |
| **T *Storage Days [% RH]** | 12.68 | 19.92 | 11.89 | <0.05 |
|  |  |  |  |  |
|  |  |  |  |  |

Supplementary Table S2 Mean values^a^ of pecan nutmeat hue (h) under different storage conditions. The replicates (n=36) consist of nutmeats from two years (2018 and 2019) and three cultivars (Stuart, Pawnee and Desirable), each having color analysis in 6 replicates for each data point. Different letters indicate significant difference between means based on Tukey’s HSD (α = 0.05).. The lowercase letters indicate differences between means at different storage times for each humidity × temperature interaction. The uppercase letters indicate difference in hue at the four humidity levels at all temperatures.

|  | **Relative humidity** | | | | | | | | |
| --- | --- | --- | --- | --- | --- | --- | --- | --- | --- |
|  | **30%** | | **50%** | | **75%** | | **80%** | | |
| **Temp (°C)** | **Days** | **Hue** | **Days** | **Hue** | **Days** | **Hue** | **Days** | **Hue** | |
| **20** | 0 | 71.48±2.79 a | 0 | 71.48±2.79 a | 0 | 71.48±2.79 a | 0 | 71.48±2.79 a | |
|  | 90 | 66.14±3.64 ab | 75 | 65.55±3.44 ab | 37 | 66.57±2.99 ab | 8 | 69.28±2.77 a | |
|  | 180 | 63.01±2.30 bc | 150 | 62.91±2.47 bc | 74 | 65.18±4.59 bcd | 24 | 68.72±2.51 ab | |
|  | 270 | 60.39±2.75 cd | 225 | 61.128±3.33 cd | 111 | 64.74±2.21 bcd | 16 | 68.52±2.83 ab | |
|  | 360 | 58.63±2.83 d | 300 | 59.35±3.96 cd | 148 | 62.66±2.73 cd | 32 | 67.73±2.48 ab | |
|  | 450 | 52.50±4.06 e | 375 | 57.77±2.93 e | 185 | 61.10±3.96 d | 40 | 65.84±2.86 b | |
|  | *Mean* | *64.24±1.94 B* | *Mean* | *64.73 ± 2.04 AB* | *Mean* | *65.64±2.38 AB* | *Mean* | | *67.52±3.28 A* |
| **30** | 0 | 71.48±2.79 a | 0 | 71.48±2.79 a | 0 | 71.48±2.79 a | 0 | 71.48±2.79 a | |
|  | 75 | 61.65±2.79 b | 45 | 66.78±2.16 b | 30 | 65.90±3.25 a | 5 | 69.60±3.06 a | |
|  | 150 | 54.78±2.98 c | 90 | 58.93±3.91 c | 60 | 57.40±6.45 b | 10 | 69.24±2.37 a | |
|  | 225 | 51.94±3.54 cd | 135 | 56.36±2.31 d | 90 | 57.11±4.01 b | 15 | 67.68±2.05 a | |
|  | 300 | 47.63±4.85 de | 180 | 53.32±2.59 de | 120 | 54.45±3.67 b | 20 | 64.71±3.03 b | |
|  | 375 | 46.75±4.60 e | 225 | 52.99±2.64 e | 150 | 52.72±3.55 b | 25 | 63.17±2.32 b | |
|  | *Mean* | *59.07±2.08 DE* | *Mean* | *61.21±2.23 CD* | *Mean* | *60.15±2.36 D* | *Mean* | | *64.25±3.39 ABC* |
| **40** | *0* | *71.48±2.79 a* | *0* | *71.48±2.79 a* | *0* | *71.48±2.79 a* | *0* | *71.48±2.79 a* | |
|  | 30 | 61.33±5.58 ab | 28 | 65.46±2.80 a | 7 | 68.20±3.03 a | 3 | 65.97±3.81 ab | |
|  | 60 | 55.93±4.52 bc | 56 | 58.81±5.91 b | 14 | 65.27±2.34 b | 6 | 62.44±2.37 bc | |
|  | 90 | 51.70±5.14 cd | 84 | 56.75±5.06 b | 21 | 60.65±2.35 c | 9 | 62.19±4.13 c | |
|  | 120 | 49.44±2.85 d | 112 | 55.33±3.31 bc | 28 | 57.29±4.06 d | 12 | 55.25±5.16 d | |
|  | 150 | 46.82±5.73 d | 140 | 50.50±5.05 c | 35 | 54.76±2.95 e | 15 | 54.32±3.49 d | |
|  | *Mean* | *56.91±2.34 EF* | *Mean* | *60.04±2.28 D* | *Mean* | *58.67±1.77 DE* | *Mean* | | *52.96±3.56 F* |

^a^ The values are tabulated as mean ± standard deviation.

Supplementary Table S3 Mean values^a^ of pecan nutmeat lightness (L) under different storage conditions. The replicates (n=36) consist of nutmeats from two years (2018 and 2019) and three cultivars (Stuart, Pawnee and Desirable), each having color analysis in 6 replicates for each data point. Different letters indicate significant difference between means based on Tukey’s HSD (α = 0.05). The lowercase letters indicate differences between means at different storage times for each humidity × temperature interaction. The uppercase letters indicate difference in lightness at the four humidity levels at all temperatures.

|  | **Relative humidity** | | | | | | | | |
| --- | --- | --- | --- | --- | --- | --- | --- | --- | --- |
|  | **30%** | | **50%** | | | **75%** | | **80%** | |
| **Temp (°C)** | **Days** | **Lightness** | **Days** | **Lightness** | | **Days** | **Lightness** | **Days** | **Lightness** |
| **20** | 0 | 72.09±4.10 a | 0 | 72.09±4.10 a | | 0 | 72.09±4.10 a | 0 | 72.09±4.10 a |
|  | 90 | 54.55±6.02 b | 75 | 55.00±6.64 b | | 37 | 51.42±4.47 b | 8 | 53.51±5.08 b |
|  | 180 | 48.83±5.61 bc | 150 | 49.57±6.15 c | | 74 | 50.79±7.13 bc | 16 | 50.17±4.40 bc |
|  | 270 | 45.99±3.68 cd | 225 | 43.73±1.29 c | | 111 | 51.27±5.41 bc | 24 | 50.88±3.97 bc |
|  | 360 | 45.07±3.25 cd | 300 | 43.72±4.44 c | | 148 | 45.22±3.68 c | 32 | 46.55±2.23 bc |
|  | 450 | 40.94±3.38 d | 375 | 43.18±3.73 c | | 185 | 45.10±3.65 c | 40 | 44.12±2.76 c |
|  | *Mean* | *56.59±2.04 A* | *Mean* | *56.46±2.05 A* | | *Mean* | *55.03±2.11 AB* | *Mean* | *48.05±2.02 D* |
| **30** | 0 | 72.09±4.10 a | 0 | 72.09±4.10 a | | 0 | 72.09±4.10 a | 0 | 72.09±4.10 a |
|  | 75 | 48.65±5.78 b | 45 | 50.11±5.51 b | | 30 | 49.79±5.32 b | 5 | 52.84±3.70 b |
|  | 150 | 44.23±4.29 c | 90 | 43.51±2.78 c | | 60 | 43.81±4.94 c | 10 | 49.82±3.35 bc |
|  | 225 | 41.06±3.46 cd | 135 | 41.64±2.01 cd | | 90 | 44.55±5.92 c | 15 | 49.27±5.16 cd |
|  | 300 | 40.31±4.97 cd | 180 | 39.93±1.98 d | | 120 | 41.05±3.98 c | 20 | 46.91±5.94 de |
|  | 375 | 39.85±3.61 d | 225 | 40.97±4.76 d | | 150 | 40.58±4.69 c | 25 | 43.83±2.79 e |
|  | *Mean* | *52.87±2.05 BC* | *Mean* | *50.70±2.09CD* | | *Mean* | *50.08±2.12 D* | *Mean* | *41.99±2.06 E* |
| **40** | 0 | 72.09±4.10 a | 0 | 72.09±4.10 a | | 0 | 72.09±4.10 a | 0 | 72.09±4.10 a |
|  | 60 | 45.52±3.85 b | 28 | 50.69±4.34 b | | 7 | 54.63±6.28 b | 3 | 49.19±4.80 b |
|  | 30 | 45.41±5.51 bc | 56 | 44.55±3.04 c | | 14 | 50.96±2.97 c | 6 | 43.03±3.14 c |
|  | 90 | 41.57±3.44 c | 84 | 44.39±3.95 c | | 21 | 46.75±3.88 d | 9 | 44.27±4.76 c |
|  | 120 | 39.63±2.95 c | 112 | 41.89±4.09 cd | | 28 | 41.65±4.20 e | 12 | 38.48±4.57 d |
|  | 150 | 38.75±3.64 c | 140 | 40.16±2.96 d | | 35 | 39.84±2.91 e | 15 | 37.40±3.41 d |
|  | *Mean* | *49.63±2.12 D* | *Mean* | | *49.93± D* | *Mean* | *42.70±2.02 E* | *Mean* | *28.10±2.36 F* |

^*^ The values are tabulated as mean ± standard deviation.

Supplementary Table S4 Mean values^a^ of pecan nutmeat chroma (C) under different storage conditions. The replicates (n=36) consist of nutmeats from two years (2018 and 2019) and three cultivars (Stuart, Pawnee and Desirable), each having color analysis in 6 replicates for each data point. Different letters indicate significant difference between means based on Tukey’s HSD (α = 0.05). The lowercase letters indicate differences between means at different storage times for each humidity × temperature interaction. The uppercase letters indicate difference in chroma at the four humidity levels at all temperatures.

|  | **Relative humidity** | | | | | | | |
| --- | --- | --- | --- | --- | --- | --- | --- | --- |
|  | **30%** | | **50%** | | **75%** | | **80%** | |
| **Temp (°C)** | **Days** | **Chroma** | **Days** | **Chroma** | **Days** | **Chroma** | **Days** | **Chroma** |
| **20** | 0 | 49.14±2.30 a | 0 | 49.14±2.30 a | 0 | 49.14±2.30 a | 0 | 49.14±2.30 a |
|  | 90 | 43.09±6.79 b | 75 | 36.50±5.21 b | 37 | 36.03±3.59 b | 8 | 34.42±3.24 b |
|  | 180 | 37.41±7.28 bc | 150 | 35.68±4.60 b | 74 | 35.85±6.68 b | 16 | 33.65±3.10 b |
|  | 270 | 35.87±5.53 bc | 225 | 31.97±2.14 b | 111 | 38.41±5.05 b | 24 | 34.95±2.47 b |
|  | 360 | 34.15±2.87 c | 300 | 34.81±4.62 b | 148 | 32.59±4.47 b | 32 | 34.41±2.17 b |
|  | 450 | 31.68±3.02 c | 375 | 33.07±2.07 b | 185 | 32.23±6.98 b | 40 | 32.34±1.91 b |
|  | *Mean* | *41.39±2.69 A* | *Mean* | *40.15±2.73 AB* | *Mean* | *39.11±2.89 AB* | *Mean* | *32.41±2.59 BC* |
| **30** | 0 | 49.14±2.30 a | 0 | 49.14±2.30 a | 0 | 49.14±2.30 a | 0 | 49.14±2.30 a |
|  | 75 | 36.14±6.13 b | 45 | 36.33±3.83 b | 30 | 35.57±3.56 b | 5 | 34.99±3.27 b |
|  | 150 | 31.72±3.16 bc | 90 | 31.32±3.41 c | 60 | 31.09±4.32 b | 10 | 33.84±2.63 b |
|  | 225 | 31.58±2.56 bc | 135 | 30.36±1.84 c | 90 | 34.14±6.02 b | 15 | 34.83±4.33 b |
|  | 300 | 29.96±4.69 c | 180 | 30.14±2.02 c | 120 | 31.22±3.70 b | 20 | 33.21±4.50 b |
|  | 375 | 30.10±3.99 c | 225 | 29.93±2.72 c | 150 | 30.11±5.49 b | 25 | 31.20±1.89 bc |
|  | *Mean* | *38.17±2.73 CD* | *Mean* | *36.56±2.85 CD* | *Mean* | *36.34±2.93 CD* | *Mean* | *29.55±2.41 D* |
| **40** | 0 | 49.14±2.30 a | 0 | 49.14±2.30 a | 0 | 49.14±2.30 a | 0 | 49.14±2.30 a |
|  | 60 | 31.22±2.57 bc | 28 | 35.95±3.35 b | 7 | 36.39±3.70 b | 3 | 34.53±4.21 b |
|  | 30 | 34.63±4.42 b | 56 | 32.90±3.29 c | 14 | 35.92±2.53 b | 6 | 28.34±2.55 cd |
|  | 90 | 31.07±2.99 bc | 84 | 33.91±3.94 bc | 21 | 35.00±2.16 b | 9 | 30.98±3.25 c |
|  | 120 | 30.09±2.20 bc | 112 | 32.66±3.08 cd | 28 | 29.96±2.31 c | 12 | 25.56±3.71 de |
|  | 150 | 29.06±5.29 c | 140 | 30.51±3.08 d | 35 | 30.16±2.37 c | 15 | 25.32±2.77 d |
|  | *Mean* | *35.46±2.93 E* | *Mean* | *36.28±2.94 E* | *Mean* | *31.03±2.52 E* | *Mean* | *18.97±2.47 F* |

^a^ The values are tabulated as mean ± standard deviation.

Supplementary Table S5 Summary of F ratio and adjusted coefficient of determination (R^2^) for the linear and exponential regression solutions for the color attributes of lightness, chroma and hue of pecan nutmeats stored under different conditions of relative humidity (RH and temperature) over different numbers of days. Data from the three cultivars Stuart, Desirable and Pawnee combined.

| **Temperature (°C)** | **RH (%)** | **Lightness** | | **Chroma** | | **Hue** | |
| --- | --- | --- | --- | --- | --- | --- | --- |
|  |  | **F ratio** | **Adj. R^2^** | **F ratio** | **Adj. R^2^** | **F ratio** | **Adj. R^2^** |
| **20** | **30** | 78.34 | 0.75 | 81.77 | 0.78 | 70.41 | 0.70 |
|  | **50** | 70.52 | 0.71 | 80.60 | 0.80 | 80.75 | 0.77 |
|  | **70** | 71.96 | 0.72 | 76.94 | 0.76 | 74.56 | 0.72 |
|  | **80** | 71.82 | 0.78 | 90.47 | 0.80 | 5.57 | 0.18 |
| **30** | **30** | 123.23 | 0.79 | 56.61 | 0.62 | 133.07 | 0.80 |
|  | **50** | 131.16 | 0.85 | 73.49 | 0.75 | 117.3 | 0.83 |
|  | **70** | 94.06 | 0.74 | 46.84 | 0.57 | 61.36 | 0.64 |
|  | **80** | 88.32 | 0.75 | 63.44 | 0.67 | 30.84 | 0.51 |
| **40** | **30** | 146.09 | 0.81 | 70.92 | 0.67 | 70.56 | 0.74 |
|  | **50** | 130.30 | 0.81 | 47.24 | 0.60 | 66.39 | 0.71 |
|  | **70** | 121.23 | 0.79 | 115.00 | 0.79 | 106.59 | 0.77 |
|  | **80** | 166.75 | 0.82 | 128.28 | 0.76 | 72.71 | 0.65 |

Supplementary Table S6 The mean values^a^ of change in hue (Δh) of pecan nutmeats with storage time in days (n=6) at different temperatures and relative humidities. Data from the three cultivars Stuart, Desirable and Pawnee combined.

|  | **Relative humidity (%)** | | | | | | | |
| --- | --- | --- | --- | --- | --- | --- | --- | --- |
| **Temp (°C)** | **30** | | **50** | | **75** | | **80** | |
|  | **Days** | **Δh** | **Days** | **Δh** | **Days** | **Δh** | **Days** | **Δh** |
| **20** | 0 | 0 | 0 | 0 | 0 | 0 | 0 | 0 |
|  | 90 | 4.69±2.53a | 75 | 4.05±1.84 a | 37 | 2.36±2.46 a | 8 | 3.24±3.13 a |
|  | 180 | 10.07±1.98b | 150 | 8.45±1.22 b | 74 | 3.49±1.46 a | 24 | 3.06±3.73 a |
|  | 270 | 12.92±1.10c | 225 | 10.35±4.08 c | 111 | 6.50±1.65 b | 16 | 3.72±3.02 a |
|  | 360 | 13.65±3.1d | 300 | 13.59±5.11 d | 148 | 9.49±1.77 c | 32 | 3.86±3.53 a |
|  | 450 | 18.03±2.65e | 375 | 15.02±1.55 e | 185 | 9.58±2.74c | 40 | 5.06±2.85 a |
| **30** | 0 | 0 | 0 | 0 | 0 | 0 | 0 | 0 |
|  | 75 | 9.94±0.89 a | 45 | 5.00±1.90a | 30 | 5.31±1.71 a | 5 | 0.803±2.03 a |
|  | 150 | 15.14±2.08 b | 90 | 13.07±4.06b | 60 | 9.00±2.71 b | 10 | 0.78±2.13 a |
|  | 225 | 19.67±1.69 c | 135 | 14.53±1.06c | 90 | 12.73±2.83 b | 15 | 3.81±0.81 b |
|  | 300 | 24.06±1.32 d | 180 | 17.67±1.89d | 120 | 16.34±3.43bc | 20 | 5.51±1.63 c |
|  | 375 | 26.28±2.11 e | 225 | 18.14±2.84d | 150 | 19.64±1.51 e | 25 | 8.07±1.62 d |
| **40** | 0 | 0 | 0 | 0 | 0 | 0 | 0 | 0 |
|  | 30 | 7.40±3.94 a | 28 | 5.38±1.52 a | 7 | 4.33±1.63 a | 3 | 3.81±1.63 a |
|  | 60 | 13.65±1.55 b | 56 | 10.16±1.54 b | 14 | 6.80±2.12 a | 6 | 8.24±2.12 b |
|  | 90 | 18.01±4.18 bc | 84 | 12.42±1.05bc | 21 | 11.03±2.00bc | 9 | 10.80±4.18 bc |
|  | 120 | 22.12±2.32 c | 112 | 14.78±1.32c | 28 | 14.59±1.52 c | 12 | 17.25±4.36 c |
|  | 150 | 22.50±4.74 bc | 140 | 18.67±2.09 d | 35 | 16.69±0.55 d | 15 | 17.73±3.36 c |

^a^The values are tabulated as mean ± standard deviation (n=6). Different letters indicate significant difference between means based on Tukey’s HSD (α = 0.05).
